# Effectiveness of Cyanoacrylate in Reducing Seroma Formation in Breast Cancer Patients Post-Axillary Dissection: A Randomized Controlled Trial

**DOI:** 10.3389/fonc.2020.580861

**Published:** 2021-01-25

**Authors:** Mahmoud Al-Masri, Fade Alawneh, Faiez Daoud, Ali Ebous, Basem Hamdan, Hani Al-Najjar, Rama Al-Masri, Marwan Abufara

**Affiliations:** ^1^ Department of Surgery, King Hussein Cancer Center, Amman, Jordan; ^2^ School of Medicine, University of Jordan, Amman, Jordan

**Keywords:** cyanoacrylate, seroma, breast cancer, breast surgery, axilla

## Abstract

**Background/Purpose:**

Seroma is a common complication after axillary dissection in women with node-positive breast cancer. We aim to determine the effect of Cyanoacrylate on reducing seroma formation in patients undergoing axillary dissection. This a randomized clinical trial.

**Methods:**

This is a single-center, randomized, single-blinded, and two-arm parallel study. Women with node-positive breast cancer eligible for axillary dissection were enrolled. Patients with a Body Mass Index (BMI) greater than 35 kg/m^2^, those who underwent immediate breast reconstruction, and/or received neoadjuvant chemotherapy were excluded. Patients were randomized in a 1:1 ratio, and were stratified according to their age, BMI, tumor size, and operation type. The primary endpoint was the total seroma volume (the total drained volume and the total aspirated volume after drain removal). Data presented as mean and range when applicable.

**Results:**

111 patients were randomized (Cyanoacrylate 57; control 54). 105 patients were analyzed. Sixty-nine patients underwent breast conserving surgery, and 36 underwent modified radical mastectomy. There was no difference in the total seroma volume between the Cyanoacrylate vs. control arms (1,304 (60–4,950) vs. 1,446 (100–5,223) ml, *p*=0.458). Wound infection, flap necrosis, number of manual aspirates, and hematoma formation were not statistically different between the two groups. Time to drain removal was shorter in the Cyanoacrylate arm (11.04(3–23) vs. 13.84(3–37) days, *p*=0.015). The use of Cyanoacrylate was not cost effective ($586.93 (550–748) vs. $29.63 (0–198), *p*<0.001). Higher seroma volume was correlated with modified radical mastectomy, older age, and BMI more than 30 kg/m^2^.

**Conclusion:**

Cyanoacrylate did not reduce seroma formation and its use was not cost effective.

**Clinical Trial Registration:**

clinicaltrials.gov, identifier NCT02141373.

## Introduction

Axillary dissection is still considered an essential procedure in the treatment of node positive breast cancer patients. Seroma formation remains the most common complication after axillary dissection with reported incidence of 15%–90% (1–6).

Although most seromas resolve within few weeks of surgery, seroma formation and its aspiration result in significant postoperative morbidity in terms of pain, discomfort, delayed wound healing, skin flap necrosis, and infection (7–9). These complications may delay adjuvant treatment and affect patient recovery along with increased financial burden on health care system.

The pathophysiology of seroma formation is not very well understood with some data implicating dead space, lymphatic leakage, and exudate as possible etiologies (10–15).

No consensus exists despite numerous suggested strategies to reduce seroma formation including drains, buttress sutures, fibrin glue or patches, tetracycline sclerosing agents, methylprednisolone, somatostatin, and shoulder exercises (13, 15–24).

The reduction of dead space after surgery is one of those strategies that can be achieved through chemical (25) and/or mechanical means (26, 27). Fibrin enriched compounds have shown seroma reduction in smaller studies, but this effect was lost in large randomized trials (23, 24, 26, 28). Surgical glue has many uses, particularly in pediatric urogenital operations, to decrease incidence of hematomas, leak, and in the treatments of lymphocele (24, 29, 30). Cyanoacrylate is a synthetic biodegradable glue (N-butyl-2-cyanoacrylate) that has shown potential for internal and external use. It has high tensile, adhesive, and hemostatic properties (31). Once it polymerizes, it creates an efficient antiseptic barrier against the most diffuse infective or pathogenic agents during surgical interventions (32).

Although Cyanoacrylate can induce a significant decrease of activated partial thromboplastin time (aPTT), no significant variations of prothrombin activity, fibrinogen, platelet number, and leukocyte cytotoxicity were identified (33). Additionally, the level of evidence to support toxicity or carcinogenicity of surgery grade Cyanoacrylate is insufficient at best (33). There were also no reports on related adverse events from the surgical use of Cyanoacrylate (30, 34, 35), this supports the safety of Cyanoacrylate in the clinical setting. Therefore, the risk of using Cyanoacrylate as surgical glue is considered minimal until new evidence suggests otherwise.

Cyanoacrylate may have the potential to reduce seroma formation after axillary dissection. The proposed mechanisms are through Cyanoacrylate’s adhesive and hemostatic properties that may impact the level and the degree of seroma formation by obliterating the dead space and creating a sealed surface to decrease lymphatic leak. The aim of this study is to investigate whether the use of Cyanoacrylate in axillary dissection reduces postoperative seroma formation.

## Methods

### Study Design

We conducted a single-center, randomized, single-blinded, and two-arm parallel study. The inclusion criteria were consenting patients aged 18 years or older who had node-positive breast cancer proven by fine-needle aspiration (FNA) or sentinel lymph node biopsy (SLNB) and were eligible for axillary dissection with or without surgical intervention for the primary tumor.

Exclusion criteria included a platelet count less than 100,000/ul, Body Mass Index (BMI) more than 35 kg/m^2^, immediate breast reconstruction surgery, patients on anticoagulation therapy or have coagulation disorders, pregnant or lactating patients, ongoing steroid therapy, prior chest radiotherapy, and patients who received neoadjuvant chemotherapy.

Post-surgery participants were excluded from statistical analysis if they developed postoperative hematoma requiring their return to the operating theatre for evacuation.

The primary outcome was the difference between the two groups in the total volume of seroma, which was calculated as the total drained volume plus the total aspirated volume after drain removal.

Secondary outcomes included safety, cost-effectiveness, time to drain removal along with the number of seroma aspirations.

The study was conducted at King Hussein Cancer Center (KHCC) Amman, Jordan, approved by the Institutional Review Board (IRB), and registered in ClinicalTrials.gov NCT02141373.

### Sample Size Calculation

A power calculation was performed before recruitment. Considering two treatment groups, testing and control group, we assumed a testing of equality where the null hypothesis is an equal means between the control and test group. Considering testing at a level of significance of 5%, powering the study at 80% and a difference of 10% or less in the means of total drained volumes (clinically irrelevant difference), 136 subjects were considered sufficient to conduct this study. However, due to slow recruitment of patients as a result of the increasing usage of neoadjuvant chemotherapy and immediate breast reconstruction (part of our exclusion criteria), an alternative approach was adopted. We reviewed the literature for likewise trials. Clement et al. conducted a multicenter, prospective, double-blinded, randomized controlled trial comparing seroma volume following mastectomy as a primary outcome. Patients were randomized into Cyanoacrylate and normal saline arms. The mean seroma volume in the control group was 1,203 ml compared to 766 ml in the Cyanoacrylate group (36). Assuming a likewise reduction in seroma volume in our study and control arms; 106 patients would be required to have an 80% chance of detecting, as significant at the 5% level, a 36% decrease in the primary outcome measure from 1,203 ml in the control group to 766 ml in the experimental group.

### Randomization and Blinding

Eligible patients were randomized in a 1:1 ratio using the randomization plan into one of the two arms: Cyanoacrylate vs. no Cyanoacrylate. The randomization process employed Excel randomization formulas and macros. The research study coordinator kept the randomization plan and informed the Operating Room (OR) manager to dispense the appropriate product accordingly. The surgeons were blinded to the patient allocation until the end of surgery when it was either required to use the product or not.

Randomization was done by Urbaniak, G. C., & Plous, S. (2013) Research Randomizer (Version 4.0) Retrieved on June 22, 2013, from http://www.randomizer.org/

### Recruitment

Between January 2014 and April 2018, 111 patients were recruited. Surgeons identified potential candidates at outpatient clinics before the planned surgery. Participants who met the inclusion criteria received a verbal explanation along with the patient information sheet. After the selection criteria were satisfied, the surgeon obtained a written informed consent. Because of the increasing role of neoadjuvant chemotherapy and the increasing number of immediate breast reconstruction (both are part of our exclusion criteria) with a parallel decrease in the number of patients eligible for axillary dissection according to the updated institutional guidelines reflecting the results from the Z011 (37), the IBCSG 23-01 (38) and the AMAROS trial (39) as standard of care, recruitment time was extended.

### Surgical Technique

All surgeries were performed by experienced breast surgeons. Axillary dissection was defined as dissection of at least level 1 and 2 axillary lymph nodes. The long thoracic and thoracodorsal nerves were routinely preserved, while the intercostobrachial nerve was preserved when feasible. Homeostasis was accomplished by knot-tie ligation or electrocautery with no vessel-sealing device allowed. Inconsistent results regarding the effect of vessel-sealing devices on seroma formation have been reported with some groups suggesting a significant increase in seroma volume compared to improved results with the use of vessel sealing (40, 41).

**Figure 1:**
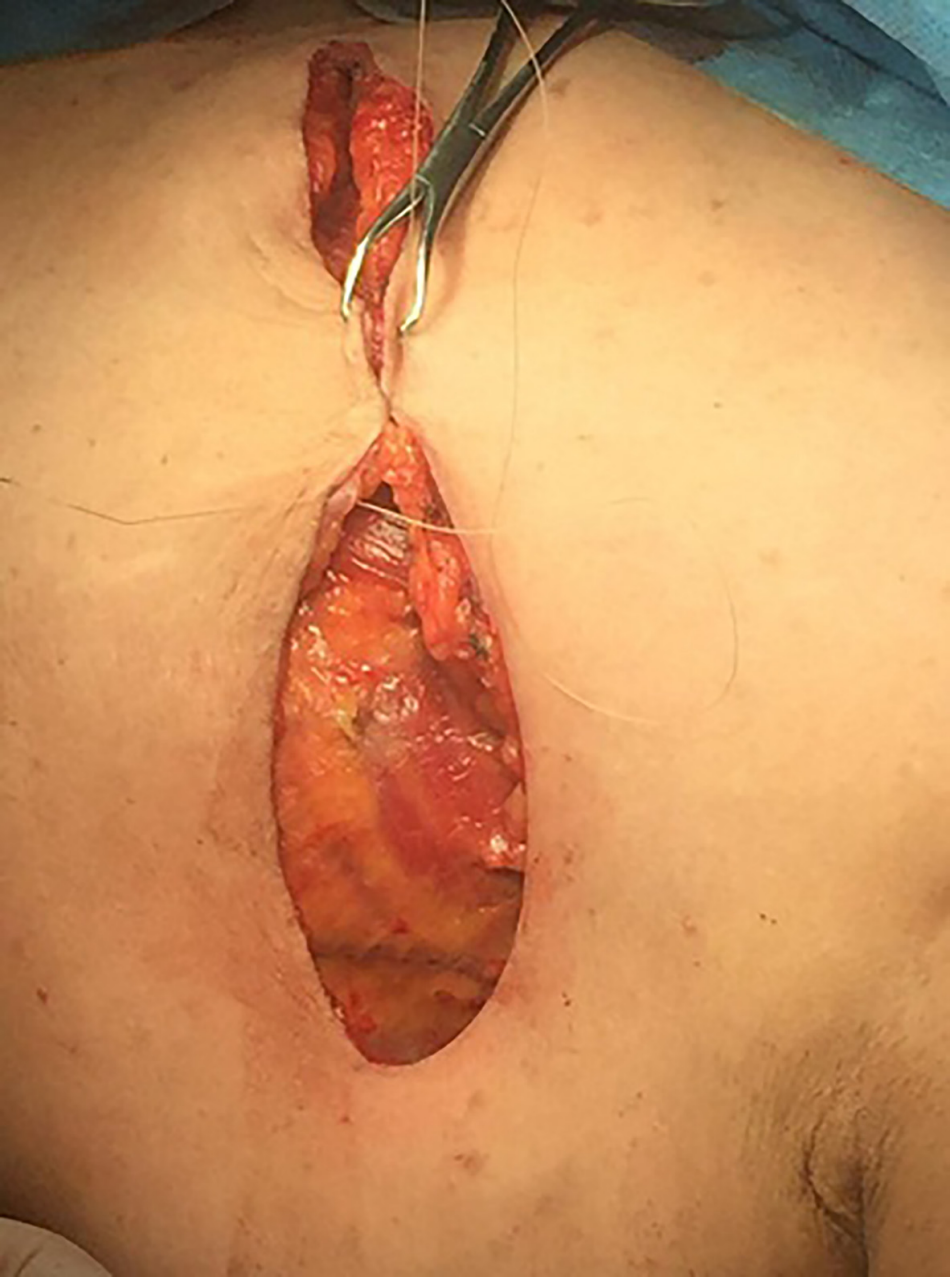
Wound closure.

**Figure 2:**
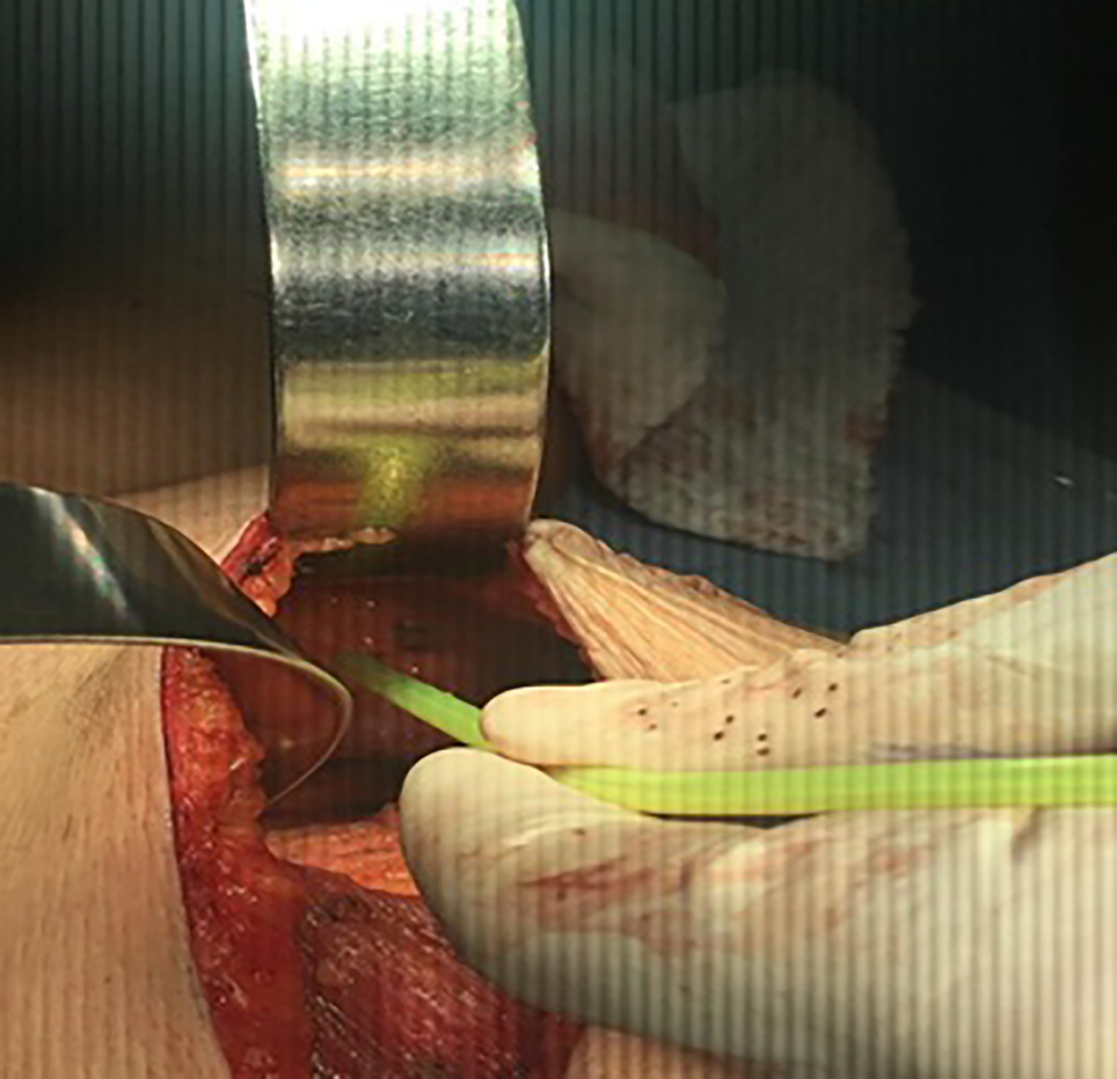
Spraying cyanoacrylate.

**Figure 3:**
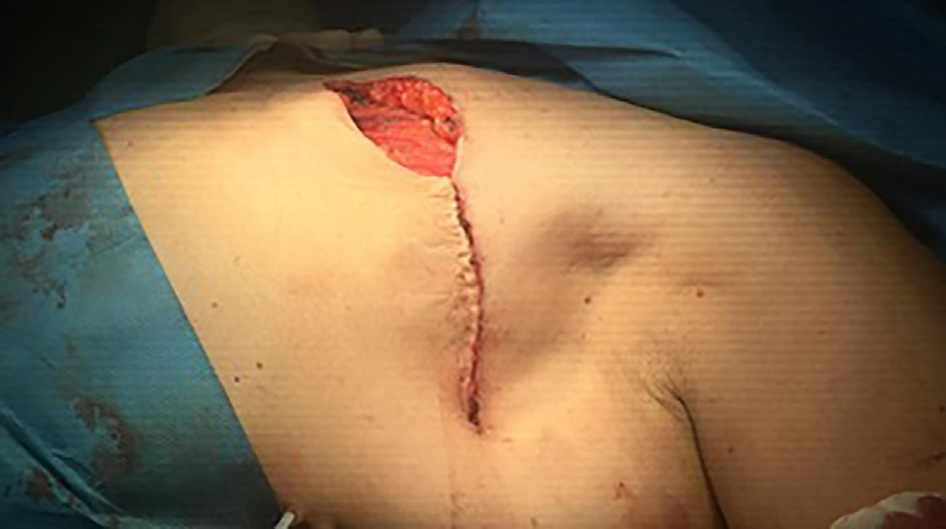
Obliteration of dead space in the axilla.

The wound was irrigated by saline solution, and one or two 16 Fr closed suction drain were placed. Wound closure was accomplished with a continuous intradermal suture line with 3/0 Vicryl (polyglactin 910) suture. In the Cyanoacrylate arm, wound closure was started medially, and 2 ml of Cyanoacrylate was sprayed into the axillary wound from a distance of 10–15 cm with a pressure-spraying device. The material was sprayed to cover the whole area dissected around the axillary vein and thoracodorsal bundle where the bulk of lymphatics in the axilla reside. A gentle compression was then applied to the chest wall for 2–3 min to allow the Cyanoacrylate to completely adhere, (**Figures 1**–**3**).

### Study Outcomes

#### Follow-Up

During the in-hospital stay, drain output was recorded by a research coordinator. At discharge, patients were instructed to begin exercising their arm 24 h after surgery.

Patients were provided with scaled bottles. They were educated to empty the drain, measure the volume of drained fluid and record it in the appropriate section of the patient diary.

Prescheduled visits into the outpatient clinic were arranged at days 5 and 14 then at 1, 2, and 3 months after the surgery at which the drain output was reviewed from the patient’s diary. The drain was removed if the output dropped to less than 50 ml/24 h.

Patients were assessed for seroma if the drain was removed. They were also assessed for signs of skin flap necrosis, wound dehiscence and wound infection. Patients who had their drain removed were instructed to visit the emergency room if they developed swelling or tension under the wound. If clinically indicated, further visits to clinic were arranged and any aspirated seroma or relevant clinical findings were registered by the treating physician.

### Statistical Analysis

Descriptive statistics were used to describe patients’ demographics such as age, BMI, T stage, N stage and operation type. Mean and range were used for the continuous variables (operation time (mins), blood loss (ml), total seroma volume (ml), aspirated seroma volume (ml), drained seroma volume (ml) and time to drain removal (days)). Patients’ demographics among the intervention vs. control groups were compared using Chi-square test.

The continuous variables (operation time (mins), blood loss (ml), total seroma volume (ml), aspirated seroma volume (ml), drained seroma volume (ml), time to drain removal (days), and the cost (USD)) were compared between the groups using t-test.

The surgical outcomes like wound infection and flap necrosis were compared using Chi-square test as appropriate.

All significant factors out of the univariate analysis were adjusted using multivariate logistic regression analysis. Odds ratio out of the model were reported.

A significance criterion of p ≤ 0.05 was considered significant and used in the analysis.

All statistical analyses of the data were carried out using IBM SPSS statistics version 24.

## Results

**Figure 4:**
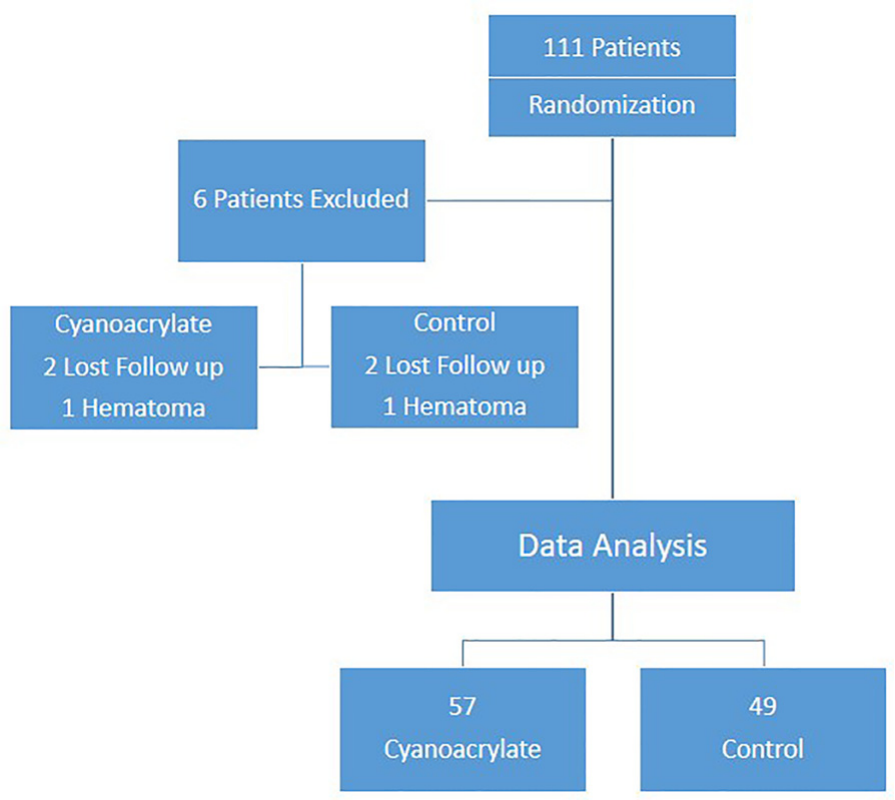
The CONSORT chart of the study.

Between January 2014 and April 2018, 111 patients were enrolled in the study. The CONSORT chart of the study is shown in **Figure 4**. Six enrolled patients were excluded, four were lost to follow up. Two developed postoperative hematoma that required their return to the operating room. Results from 105 patients (56 in the Cyanoacrylate arm and 49 in the control arm) were subjected to statistical analysis.

The groups were comparable in terms of age, BMI, clinical T and N stages, and the frequency of breast-conserving surgery versus mastectomy (**Table 1**).

**Table 1:** Patients’ clinical and pathological characteristics.

	Value	All patients (*n* = 105)	Cyanoacrylate (*n* = 56)	Control (*n* = 49)	*P* Value
Patient age	<50	39	25	14	0.157
	50-65	40	17	23	
	≥65	26	14	12	
BMI	<30	51	29	22	0.481
	≥30	54	27	27	
T Stage	Tis	2	1	1	0.230
	1	26	18	8	
	2	62	32	30	
	3	14	5	9	
	4	1	0	1	
N Stage	1	57	32	25	0.786
	2	29	15	14	
	3	19	9	10	
Operation	BCS	69	38	31	0.621
	Mastectomy	36	18	18	
Operation Time (mins)	Mean	–	138	135	0.726
	Range		(80-320)	(65-200)	
Blood Loss (ml)	Mean	–	95	77	0.775
	Range		(20-500)	(10-300)	

BMI, body mass index; BCS, breast conserving surgery.

**Figure 5:**
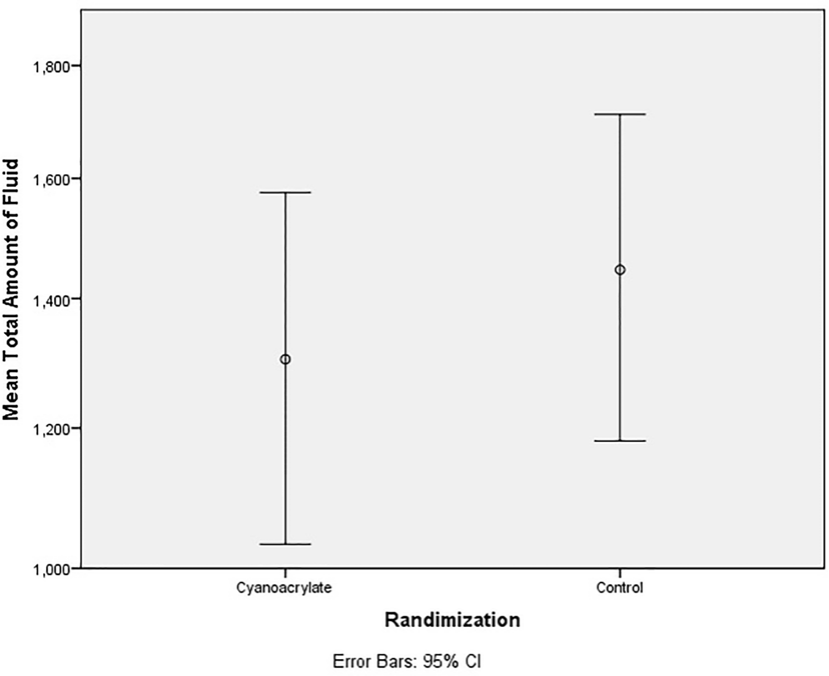
Dot plot; Mean Total Amount of Fluid (ML).

**Figure 6:**
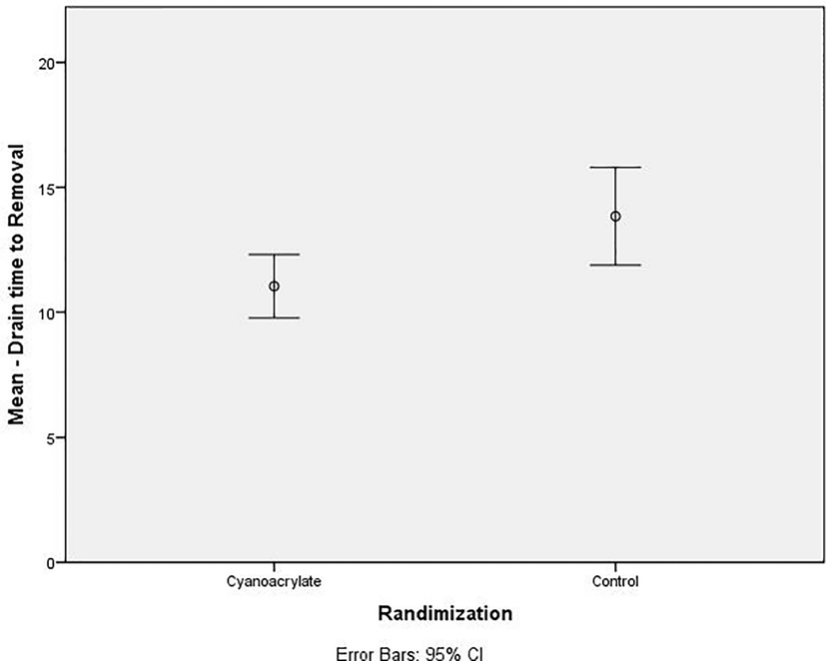
Dot Plot; Drain to time removal (Days).

The use of Cyanoacrylate did not affect the total volume of seroma with a mean value of 1304.68 ml versus control group 1,446.51 ml, *p=*0.548 (**Table 2**), (**Figure 5**). The time to drain removal was significantly shorter in the Cyanoacrylate group with a mean of 11.04 days in the Cyanoacrylate arm vs. 13.84 days in the control arm, *p*=0.015 (**Table 2**), (**Figure 6**). There was no difference in the incidence of either wound infection or flap necrosis between the two study arms. By calculating the total cost including manual aspiration, clinic visit, and Cyanoacrylate cost; the use of Cyanoacrylate was associated with a significantly higher cost compared to the control arm with a mean of $586.93 in the Cyanoacrylate arm vs. $29.63 in the control arm, *p*=<0.001 (**Table 2**).

**Table 2:** Outcomes.

	Value	All patients (n = 105)	Cyanoacrylate (n = 56)	Control (n = 49)	*P* Value
Total Seroma Volume (ml)	Mean Range	1,370.87(60–5,223)	1,304.68(60–4,950)	1,446.51(100–5,223)	0.548
Aspirated Seroma Volume (ml)	MeanRange	240(0–1,890)	259.11(0–1,890)	218.16(0–1,810)	0.627
Drained Seroma Volume (ml)	MeanRange	1,130.87(50–5,223)	1,045.57(60–4100)	1,228.35(50–5,223)	0.255
No. of Manual Aspiration	MeanRange	1.52(0–9)	1.68(0–9)	1.35(0–9)	0.429
Time to Drain Removal (Days)	MeanRange	12.34(3–37)	11.04(3–23)	13.84(3–37)	0.015
Wound Infection	YesNo	699	254	445	0.312
Flap Necrosis	YesNo	4101	155	346	0.247
Cost (USD)	MeanRange	326.86(0–748)	586.93(550–748)	29.63(0–198)	< 0.001

Univariate and multivariate analyses of variables influencing the total seroma volume included age, BMI, type of surgery (mastectomy vs breast-conserving surgery (BCS)), number of lymph nodes harvested in addition to Cyanoacrylate use (**Table 3**). Higher seroma volume was independently associated with a BMI greater than 30 kg/m^2^, older age, and mastectomy rather than breast-conserving surgery (BCS) (**Table 4**). The number of lymph node harvested, and Cyanoacrylate use did not affect the total volume of seroma (**Table 3**).

**Table 3:** Univariate Analysis of factors influencing total seroma volume.

	Value	*P* Value
Age	<50	< 0.001
	50–65	
	≥65	
BMI	<30	0.004
	≥30	
Operation Type	Mastectomy BCS	0.043
Lymph Nodes Harvested	11–20	0.726
	21–30	
	31–40	
	41–50	
Randomization	Cyanoacrylate Control	0.458

BMI, body mass index; BCS, breast conserving surgery.

**Table 4:** Multivariate Analysis of factors influencing total seroma volume.

	Value	Odd Ratio	*P* Value
Age	<50	16.1	< 0.001
	50–65		
	≥65		
BMI	<30	16.1	0.004
	≥30		
Operation Type	Mastectomy BCS	4.2	0.043

BMI,body mass index; BCS,breast conserving surgery.

## Discussion

In the current trial, the use of Cyanoacrylate in patients undergoing axillary dissection didn’t affect seroma formation but was associated with earlier drains removal. No increase in the incidence of flap necrosis or wound infection were noted even in patients with high risk of seroma formation (defined as BMI > 30, age > 60 and those undergoing mastectomy), (**Table 4**). And the use of Cyanoacrylate in this high risk group did not affect seroma formation (**Table 5**).

**Table 5:** High Risk Group Analysis of factors influencing total seroma volume.

	Value	Cyanoacrylate Mean (Std.Div)	Control Mean (Std.Div)	*P* Value
Age	≥60 years	2,048.78(1172.37)	1,604.45(811.59)	0.166
BMI	≥30	1,649.00(1101,60)	1,614.11(1007.29)	0.904
Operation Type	Mastectomy	1,062.72(718.73)	1,148.67(812.21)	0.739

BMI, body mass index.

Axillary surgery in the form of axillary dissection is the standard of care in the management of node positive axilla. Although major changes emerged to minimize the extent of surgical intervention in the axilla. Axillary dissection is associated with significant morbidity with seroma formation being the most common ranging from 15-90% (1–6). This may delay adjuvant treatment and affect patients’ recovery along with increased financial burden on health care systems. Various methods have been tested to decrease seroma formation either by obliterating the dead space or sealing the lymphatics with no consensus on a best single method.

Our study identified BMI more than 30 kg/m^2^, age greater than 60, and mastectomy compared to BCS as independent predictors of higher volume seroma. Those findings correspond with those reported by others (42–44). A linear association between increasing BMI and seroma formation in breast surgery may be explained by the tendency of adipose tissue to culminate in higher exudate rate (45). In addition, older age group was associated with higher level of seroma due to the possible influence of senile changes on lymphatics and capillaries.

Neoadjuvant chemotherapy and breast reconstruction were considered part of exclusion criteria because of the conflicting evidence regarding their role in seroma formation. In addition, breast reconstruction changes in the axillary dissection pocket geometry after reconstruction (5, 42, 43, 45).

To the best of our knowledge, only two randomized controlled trials addressed the use of Cyanoacrylate in breast cancer surgery. The results from these trials are conflicting.

The study from Greece by Kalliopi et al. (44) detected a significant reduction in seroma formation, duration of drainage, and amount of drainage with the use of Cyanoacrylate. In this trial, 128 women with breast cancer were scheduled for a modified radical mastectomy or quadrantectomy with lymph node dissection. Cyanoacrylate adhesive was applied to the operative field after the removal of the tumor and lymph nodes (n=64 patients) while controls received saline (n=64 patients). The distribution of the type of surgery in each arm was not stated. The authors recommended the use of Cyanoacrylate for patients with high risk of seroma formation after surgery for breast cancer (44).

A study from Australia by Clement et al. (36) compared the use of Cyanoacrylate versus normal saline during the wound closure in participants (n=76 patients) undergoing mastectomy with or without axillary dissection. The trail showed no benefit to the use of Cyanoacrylate in mastectomy and axillary surgery as far as reduction in the risk of seroma formation was concerned. Moreover, in elderly and obese participants, the use of Cyanoacrylate showed an increase in seroma formation and postoperative wound infection. The results described by Clement et al. are in consistent with our observations. No added benefit was found for Cyanoacrylate in decreasing seroma after breast cancer surgery. In our study, Cyanoacrylate did not show significant reduction in seroma formation for the overall study population as well as the high-risk groups identified. When comparing the seroma volume to control arm for this subset of patients, it was not statistically different.

For the time being, the only effective way that can decrease the morbidity associated with axillary dissection is to minimize surgical intervention in the axilla, further studies are needed to establish the role of Cyanoacrylate in breast cancer surgery.

## Conclusion

Cyanoacrylate use in axillary dissection did not affect seroma formation and its usage in axillary dissection was not cost effective.

## Data Availability Statement

The original contributions presented in the study are included in the article/supplementary materials. Further inquiries can be directed to the corresponding author.

## Ethics Statement

The studies involving human participants were reviewed and approved by Dr. Maysa Al-Hussaini Chairperson, Institutional Review Board at the King Hussein Cancer Center. Written informed consent to participate in this study was obtained from all participants.

## Author Contributions

MA-M, FA, FD, AE, BH, HA-N, RA-M, and MA contributed to the design and implementation of the research, to the analysis of the results, and to the writing of the manuscript. All authors contributed to the article and approved the submitted version.

## Funding

This study was funded by King Hussein Cancer Center (Grant Number: 12KHCC54).

## Conflict of Interest

The authors declare that the research was conducted in the absence of any commercial or financial relationships that could be construed as a potential conflict of interest.
